# Effect of *Bacillus amyloliquefaciens* supplementation on intramuscular fat accumulation and meat quality in finishing pigs

**DOI:** 10.5713/ab.24.0399

**Published:** 2024-10-25

**Authors:** Thami Wiseman Ndlandla, Fu Yuan Cheng, Chao Wei Huang, Kuo Tai Yang

**Affiliations:** 1 Department of Tropical Agriculture and International Cooperation, National Pingtung University of Science and Technology, Pingtung 912301, Taiwan; 2 Department of Animal Science, National Pingtung University of Science and Technology, Pingtung 912301, Taiwan

**Keywords:** *Bacillus amyloliquefaciens*, Carcass Traits, Finishing Pigs, Gene Expressions, Intramuscular Fat, Meat Quality

## Abstract

**Objective:**

This study evaluated the potential of *Bacillus amyloliquefaciens* to improve growth performance and meat quality in finishing pigs.

**Methods:**

Thirty-two female Landrace×Duroc pigs, 21 weeks old with initial body weight 77.45±3.29 kg, were divided into two groups: a control group (basal diet) and a probiotic group (basal diet with *Bacillus amyloliquefaciens* at 1×10^9^ CFU/kg). Body weight and average daily gain (ADG) were recorded at the start and at fortnight intervals for a 56-d feeding trial. At the end of the experiment, carcass traits, meat quality and intramuscular fat related gene expression of *longissimus dorsi* muscle were analyzed.

**Results:**

The probiotic group showed significantly higher final body weight and D0–D56 ADG (p<0.05). Additionally, the probiotic group had greater carcass weight, back fat thickness and marbling score (p<0.05), while the lean meat percentage remained unchanged. Meat quality analysis revealed that the probiotic group had a higher b* value (5.47) (p<0.05), and a lower shear value (p<0.001), but there was no effect on the a* value and water holding capacity. Moreover, probiotic treatment increased the gene expression of fatty acid uptake and regulators, such as lipoprotein lipase (LPL), cluster of differentiation 36 (CD36), and solute carrier family 27 member 1 (SLC27A1) (p<0.05).

**Conclusion:**

Our findings suggest that the supplementation of *Bacillus amyloliquefaciens* not only enhanced growth performance and carcass weight in finishing pigs, but also improved marbling and tenderness in the *longissimus dorsi* muscle through the up-regulation of lipogenic-genes related to fat accumulation. This indicates its potential as feed additive to enhance pork quality.

## INTRODUCTION

Pork is the most consumed animal protein worldwide, which takes account for 33% of total meat consumption. Based on the market demand, the most common system for pricing pork is to use lean meat percentage (LMP) [1]. However, due to the paradigm shift, the consumer preference is changing from leanness to fatness. Pigs with higher marbling are considered premium by consumers due to their fat content. The most attributes of premium pork include tenderness, juiciness, flavour and absence of off-flavours. In addition, increasing the intramuscular fat (IMF) or marbling level also improves the acceptability of pork. Previous study showed positive relationships between acceptability, tenderness and the level of IMF content, with all the consumers preferring loins with higher IMF levels [2]. From the pig production perspective, IMF and backfat thickness are highly correlated with the eating quality traits of meat, such as juiciness, flavor, and tenderness [3,4]. In addition, higher level of marbling or IMF has a positive influence on the sensory experience and also improves the acceptability of pork, but if the level is over 3.5% that will increase the rejection due to the visible fat [5,6]. Hence, the producers are trying to utilize feed additives to promote IMF accumulation for premium meat production.

Probiotics have been widely used for different purposes in pig industry, such as improving gut health, immunity, growth performance and meat quality [7–9]. According to Tian et al [10], dietary probiotic supplementation (*Lactobacillus reuteri 1*, *LR1*) improved pork quality compared to antibiotics by decreasing drip loss and shear force. Ganeshkumar et al [11] reported that commercial probiotic feed (*Biobloom*) could significantly enhanced carcass traits and sensory quality of pork in large scale swine production. In another study, Dowarah et al [12] demonstrated that supplementing a basal diet with the probiotic *Lactobacillus* (NCDC-15) improved growth performance, fecal microbial count, and intestinal morphology in grower-finisher pigs. Moreover, Balasubramanian et al [13] supplemented the basal diet of pigs with a multi-species probiotic (MSP), which resulted in increased body weight, average daily gain (ADG), and gain-to-feed ratio, without affecting feed intake. The probiotics also improved sensory evaluations of color and backfat thickness, and tended to increase firmness while reducing cooking loss and drip loss. These results suggested that dietary MSP positively impacts both the growth performance and meat quality of pigs. Zhu et al [14] reported that dietary probiotics supplementation on sows and offspring piglets increased IMF content in the psoas major muscle (PMM) at 95 days old, polyunsaturated fatty acid (PUFA) and n-3 PUFA levels in the *longissimus dorsi* muscle (LDM) at 65 days old, C16:1 levels in the LDM at 125 days old. In addition, utilizing *Bacillus*-based probiotics improved growth via improving the apparent total tract digestibility of crude protein (CP), fat and phosphorus (P) [15]. The combination of *Bacillus amyloliquefaciens* and *Bacillus subtilis* has also been shown to improve the feed conversion ratio in growing-finishing pigs [16].

Even though the studies show the benefits of probiotics in improving the growth performance and meat quality in pigs, limited research addressed the specific function of single strains of *Bacillus amyloliquefaciens* in growing-finishing pigs. Therefore, this study investigated the effects of *Bacillus amyloliquefaciens*, on growth performance and carcass traits of finishing pigs, and determined the meat quality and degree of IMF deposition through associated gene expression in *Longissimus dorsi* (LD) muscle of pigs.

## MATERIALS AND METHODS

### Animal care

The animal care protocols were conducted in compliance with the guidelines set forth by the Institutional Animal Care and Use Committee (IACUC) of the General Research Service Center, National Pingtung University of Science and Technology, Taiwan (Protocol No: NPUST-111-007).

### Animals, housing, and treatments

A total of thirty-two female crossbred (Landrace×Duroc) finishing pigs, with an average weight of 77.45±3.29 kg, were randomly divided into two treatment groups, each comprising 16 pigs, with 8 pigs housed per pen. Two groups were basal diet (control group) and basal diet supplemented with *Bacillus amyloliquefaciens* at 1×10^9^ CFU/kg (probiotics group). In this experiment the *Bacillus amyloliquefaciens* was obtained from BiomiXin Co., Ltd, Pingtung, Taiwan. The composition of the experimental diets is presented in Table 1. Pigs had *ad libitum* access to the feed and drinking water. The Individual pig body weights were recorded at the initial point (day 0) and subsequently every 14 days throughout the experimental period. These measurements were used to calculate growth parameters, including ADG [7]. After 56 days, the live weights of pigs were measured before transportation to the slaughterhouse. Eight pigs from each group were selected for further carcass analysis based on the average body weight in each pen. Commercial procedures were employed for the slaughter process in a federally inspected facility. The live weights of pigs were recorded immediately before slaughter, and hot carcass weights were measured approximately 1-hour postmortem.

### Carcass characteristics and sample collection

Carcass traits were evaluated 24 h post-mortem. Back-fat thickness (cm) was measured by determining the average vertical thickness of the vertebrate of the 1st and last rib, and the last lumbar vertebrate from the edge of the connective tissue to the outer layer of fat, with modifications from the protocol described by Song et al [17]. A cross-sectional surface incision was made at the 10th and 11th ribs of the LD muscle, and the area was measured using an area meter (KP-90; Koizumi Seiki Co., Osaka, Japan) to determine loin eye area (cm^2^), following slight modifications from the protocol by Pringle and Williams [18]. Lean percentage was calculated as the percentage of lean weight to carcass weight. The calculation involved determining the amount of lean meat trimmed by zero fat specification, plus tendon meat of the limbs and the whole lean meat of the trimmed tendon meat, the trimmed minced lean meat after trimming, plus the tendon meat of the limbs and the trimmed minced lean meat, divided by the slaughter weight, with modifications from the method described by Song et al [17]. The marbling score was measured or rated according to the protocol by Qiao et al [19]. A thin chop, approximately 5.0 cm thick, was removed from the LD muscle at the 10th rib and used for all of the subsequent measurements and analysis. Samples were packed in polyethylene bags and refrigerated in a cold chamber at 4°C to subsequent measure of meat quality, including muscle pH_48h_, color, cooking loss, drip loss, water-holding capacity (WHC), and shear force.

### Meat quality assessment

Post-mortem pH value at 48 h was measured using a portable pH meter (Model SP-2100; Suntex Instruments, New Taipei, Taiwan) connected with a pH electrode. A total of 5 g meat sample was minced, added to 50 mL distilled water, and homogenized for 1 min to determine the pH value, following the method outlined by Troutt et al [20].

Meat color CIE Lab parameters (L* = lightness, a* = redness, and b* = yellowness) were measured after blooming for 20 min by taking six measurements on each side of the sample using a hand-held colorimeter (CR-410; Konica Minolta Inc., Tokyo, Japan). The mean value of the six measurements was used as the result.

For drip loss measurement, meat samples were cut into pieces (1 cm×1 cm×2 cm) and weighed. Then, the trimmed pieces were hung in polyethylene bags for 24 h at 4°C. The percentage of weight loss to initial weight was recorded as the drip loss (%) as described by Song et al [17].

For cooking loss measurement, LD samples were wiped dry of surface moisture, weighed (W1), wrapped in aluminum foil, and cooked in a water bath at 80°C for 40 min to bring the core temperature above 75°C. After cooling to room temperature, the samples were weighed again (W2) to calculate cooking loss as the percentage of weight before cooking (W1) minus weight after cooking (W2), relative to the weight before cooking according to Guignot et al [21].

WHC was determined with slight modifications from the method described by Musa et al [22]. A 1 g block of pork sample was placed on a filter paper (No.1, ADVANTEC; Tokyo, Japan), covered with acetate paper, and pressed at 3000 psi for 60 seconds using a Carver Press (Wabash, Indiana, USA). Water retention was calculated as the ratio of the surface area of the meat to the total surface area.

Shear force values were measured following the method by Alonso et al [23] using the Physical Property Tester (TA.XT Plus; Stable Micro System, Godalming, UK) linked to a computer system. The measurement conditions included a measurement probe Warner–Bratzler shear blade tool with a downward pressure depth of 30 mm, and a probe drop speed of 1 mm/sec. Pork samples were trimmed to specifications (5 cm×1 cm×1 cm) after cooking and cooling at room temperature before measuring the shear force values.

### Intramuscular fat accumulation

The IMF content was measured following the method outlined by Holman et al [24] using Soxhlet extraction. This involved using an extraction unit (ST 225 Soxtec; FOSS Analytical Solutions Pty. Ltd., Victoria, Australia). Approximately 2.5 g of freeze-dried sample was placed in a porous thimble and extracted in 86 mL of hexane for 60 min within individual extraction tins. The residues were allowed to evaporate for an additional 20 minutes, then placed in a forced draft oven for 30 min at 105°C to remove any residual solvent. The difference in sample weight before and after extraction was used to calculate crude fat content, expressed as a percentage of the fresh (wet) sample weight.

### Gene expression analysis

Total RNA was isolated from the 10th rib muscle of the pigs using the TRIsure (Bioline, London, UK), followed by conversion to complementary DNA (cDNA) using the MMLV Reverse Transcription Kit (Protech Technology Enterprise Co., Ltd., Taipei, Taiwan) according to the manufacturer’s instructions. The extracted RNA was immediately placed in liquid nitrogen and stored at −80°C until further use. RNA extraction was performed using the Total RNA Extraction Kit (Gibco, Waltham, MA, USA) following the manufacturer’s protocol. Subsequently, reverse transcription polymerase chain reaction (RT-PCR) was conducted to measure mRNA gene expression levels, including lipoprotein lipase (LPL), cluster of differentiation 36 (CD36), fatty acid binding protein 3 (FABP3), fatty acid binding protein 4 (FABP4), solute carrier family 27 member 1 (SLC27A1), patatin like phospholipase domain containing 2 (PNPLA2), peroxisome proliferator-activated receptor gamma gene (PPARγ), fatty acid synthase (FASN), preilipin 2 (PLIN2), and carnitine palmitoyltransferase 1β (CPT1β). Real-time quantitative polymerase chain reaction (qPCR) analysis was in a 10 μL of mixture containing 5 μL of 2X SYBR Green Master Mix, 1 μL of cDNA (50 ng/μL), 0.4 μL of 10μM forward and reverse primer, and then carried out on the Step-One Plus Real-Time PCR System (Applied Biosystems, Waltham, MA, USA). After a single pre-denaturation cycle at 95°C for 1 min, followed 40 cycles (95°C for 15 s, 60°C for 1 min) amplification and a melting curve program. The expression levels were calculated using the 2^−ΔΔCt^ method, with the glyceraldehyde-3-phosphate dehydrogenase (GAPDH) gene used for normalization. The primer sequences for quantitative real-time qPCR are provided in Table 2.

### Statistical analysis

Statistical analysis was conducted using the Statistical Package for the Social Sciences (SPSS) software version 20.0. The independent sample t-test was employed to compare the measured data between the experimental treatment groups, comprising 32 and 16 animals for growth performance and carcass quality, respectively. A significance level of p<0.05 was considered indicative of a statistically significant difference in the results.

## RESULTS AND DISCUSSION

### Growth performance

Probiotic supplementation significantly improved the growth performance of finishing pigs, as evidenced by the notable increase in body weight and ADG (p<0.05) (Table 3). During the finishing period, the body weight of pigs in the probiotic group at days 42 and 56 was significantly higher than the body weight of pigs in the control group at the same time points. Meanwhile, the ADG showed no statistical differences among the groups except during days 29 to 42 (p<0.05). Notably, the ADG was significantly higher throughout the entire experimental period in the probiotic group (p<0.001). These findings align with previous studies, which indicated that dietary supplementation with probiotics could increase body weight and improve growth performance in both piglets and finishing pigs [13]. The benefits of probiotics in enhancing growth performance and feed efficiency may be attributed to improved digestibility and modulation of intestinal microflora [25].

### Carcass traits

Compared with the control group (Table 4), dietary supplementation with probiotics significantly increased the carcass weight of pigs (p<0.01). Supplemental probiotics also significantly increased back fat thickness and marbling scores of finishing pigs (p<0.05). However, there were no significant differences in LMP, fat percentage, loin eye area, and bone percentage. Importantly, the primal retail cuts of pig carcass, including shoulder loin, loin, belly, tenderloin and ham were unaffected by dietary probiotic treatment (Table 4). Previous studies have highlighted the significant benefits of feeding probiotics to pigs. Balasubramanian et al [13], van der Peet-Schwering et al [16] and Ganeshkumar et al [11] reported that carcass characteristics are directly proportional to the body weight at the time of pigs’ slaughtering. Samac et al [26] observed a statistically significant rise in backfat thickness in pigs as their body weight increased, and suggested that heavier pigs are associated with a rise in the absolute proportions of the ham, loin, chin, adipose tissue, and less valuable parts. Furthermore, Hoa et al [27] observed a positive correlation between backfat thickness and IMF in the LD muscle, indicating that an increase in backfat thickness or fat deposition leads to a corresponding increase in IMF in the LD muscle. In the current study, the probiotics group, characterized by higher body weight, carcass weight, backfat thickness, and marbling score, numerically increase fat percentage but there was no decrease in lean percentage. Despite the increased fat content, the percentages of primal cuts, such as loin, belly, tenderloin, and ham, remained unaffected, preserving crucial determinants of carcass quality. From an economic perspective, supplementing *Bacillus amyloliquefaciens* in pig diets not only benefits carcass weight but also maintains the characteristics of valuable primal cuts, aligning with consumer preferences.

### Meat quality

Based on the meat quality parameters (Table 5), probiotics supplementation had no significant effect on pH, L* and a* value (p>0.05). A significant increase of b*value in probiotics group was observed in the LD muscle (p<0.01). However, probiotics had no significant effect on cooking loss, drip loss, and WHC of finishing pigs, but decreased shear force in the probiotics group (p<0.05). Generally, increasing backfat thickness increased L* (lightness) and decreased a* (redness) values of LD muscle. Ba et al [28] indicated that higher slaughter weight was associated with increased fat and moisture content, decreased cooking loss and higher WHC. However, they disagreed with our results. Nevertheless, the findings of Chang et al [29] was consistent with our observation, as they reported a significant increase in b*value and decrease in shear force of LDMs of pigs fed a probiotic-supplemented diet (*Lactobacillus plantarum*; 2.2×10^8^ CFU/mL). This was attributed to a due to decreased myofiber diameter and cross-sectional area of the muscle, which may improve WHC and pork tenderness, resulting from desirable changes in muscular morphology.

The IMF content, determined by measuring crude fat, increased with probiotic supplementation. The probiotic group exhibited higher IMF content (6.26) compared to the control (5.02), but there was no statistical difference between the groups (p>0.05). IMF content is considered a crucial indicator of meat quality, particularly in terms of eating quality and consumer preference. Sladek and Mikule [30] reported that IMF content varies with the body weight of pigs. Specifically, pigs with a body weight range of 110 to 119.9 kg had higher IMF content, while those with a body weight below 100 kg had lower IMF content. These findings indicated that pigs with an average final body weight of approximately 120 kg exhibited higher IMF content, which is consistent with the results of our study. Furthermore, Latorre et al [31] and Samac et al [26] found that back fat thickness and dressing percentage increased significantly with an increase in body weight of pigs. As previously mentioned, probiotic supplementation resulted in statistically higher back-fat thickness and marbling score, alongside numerically increased IMF deposition, but the increase was not statistically significant. Our results were consistent with those of Hao et al [32] and Ganeshkumar et al [11] who observed an increase of IMF and marbling score after supplementation with fermented mixed feed and a commercial probiotic Biobloom 5 g / pig / day, respectively. In another experiment, Niu et al [33] demonstrated a direct correlation between high IMF deposition and marbling score, which improved pork tenderness. Tenderness, which is one of the most important meat trait for consumer, can be quantified by shear force [34]. Our study demonstrated that the administration of *Bacillus amyloliquefaciens* significantly improved meat tenderness, as evidenced by a reduction in shear force. Daszkiewicz et al [35] evaluated the influence of varying IMF content (≤1.0%, ≤1.01% to 2.0%, ≤2.01% to 3.0%, and >3.0%) on quality of pork. The sensory analysis conducted by Daszkiewicz et al [35] indicated that pork palatability, juiciness, and tenderness improved with IMF content above 3.0%. The study further suggested that the positive effects of higher IMF content on the sensory qualities of meat may be observed most pronounced within a concentration range of 2.5% to 3.5%. Our study corroborates these findings, revealing that higher IMF content was associated with a significantly higher marbling score, reduced shear force, thereby significantly enhancing meat tenderness. These results suggest that probiotics like *Bacillus amyloliquefaciens* could be beneficial as feed additives in improving meat quality in pigs.

### Gene expression analysis

**Figure 1:**
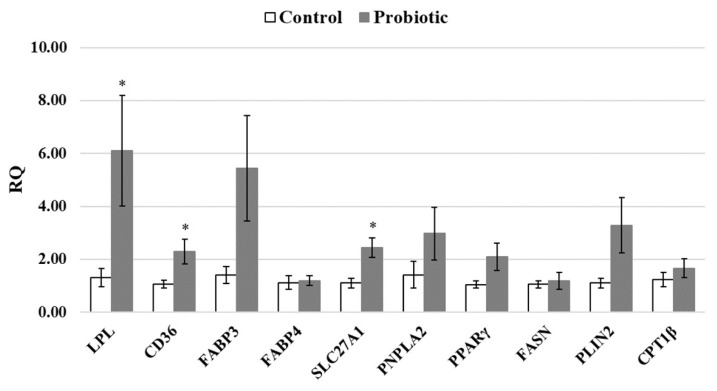
Relative expression level of transcripts related to lipid metabolism genes in the *Longissimus dorsi* muscle after probiotic supplementation in finishing pigs. RQ, relative quantitation; LPL, lipoprotein lipase; CD36, cluster of differentiation 36; FABP3, fatty acid binding protein 3; FABP4, Fatty acid binding protein 4; SLC27A1, solute carrier family 27 member 1; PNPLA2, patatin-like phospholipase domain containing 2; PPARγ, peroxisome proliferate-activated receptor gamma; FASN, Fatty acid synthase; PLIN2, preilipin 2; CPT1β, carnitine palmitoyltransferase 1β. * Significantly different compared with the control group at p<0.05 (n = 8).

Based on the previous study, we already observed the effect of probiotics that could increase the backfat thickness and lower the shear force (Tables 4, 5). Even though many studies addressed the genes that related to IMF formation, limited study links the probiotics with IMF formation. To investigate the lipid metabolism related genes that affect the fat tissue and IMF formation, we utilize the candidate genes that related to the pathway of IMF formation, which includes lipid uptake, lipogenic genes and lipid oxidation. Results revealed that supplementation of probiotics markedly increased the gene expression of fatty acid uptake and regulator related genes, including LPL, CD36, and SLC27A1 (p<0.05) (Figure 1). Additionally, the expression levels of lipogenic gene in the cells, which including PNPLA2, PPARγ, and PLIN2 were higher than that of control group without statistical difference (p>0.05). Numerous studies demonstrated that meat tenderness was related to increases of IMF content as a consequence of regulated fatty acid metabolism in muscle [10,36]. Our results suggest that supplementation of probiotics upregulated the lipogenic rates by increasing the lipid uptake (LPL, CD36 and SLC27A1), and lipogenic effect in cells (PPARγ and PLIN2). Moreover, the expression of CD36 and SLC27A1 are positively associated with long-chain fatty acids uptake into differentiating adipocyte. These results were consistent with Wang et al [37] who reported the increase fat deposition and decrease in fat removal, thus contributing to increase IMF content in the LD muscle of Laiwu pigs. Kim et al [38] reviewed the relationship between pig slaughter weight and carcass characteristics, noting a faster rate of fat deposition compared to protein during the late finishing period. This resulted in significantly greater carcass fat content in pigs with heavier market weights. In the present study, dietary probiotics improved pig growth performance, resulting in increased carcass weight and fat accumulation in the LD muscle, which contributed to enhanced meat tenderness. Additionally, the probiotics group exhibited increased carcass weight and back fat thickness without altering fat percentage, potentially increasing retail value. *Bacillus amyloliquefaciens* have been reported to increase insulin-like growth factor (IGF)-1 to improve lipogenesis by regulating the Firmicutes/ Bacteroidetes ratio that affect the fat deposition [39]. However, further research is warranted to elucidate the underlying mechanisms of how *Bacillus amyloliquefaciens* regulate the lipogenic gene in muscle, especially the value primal cuts meat.

## CONCLUSION

The study indicates that supplementation of *Bacillus amyloliquefaciens* probiotics in finishing pigs was beneficial in increasing the growth performance, carcass weight, and marbling score. Additionally, meat quality was improved as indicated by low shear force values due to more IMF deposition. Moreover, probiotic supplementation upregulated the mRNA levels of key regulatory genes for fat deposition in muscle tissue of pigs. Therefore, the results provide new insights into how probiotics can improve the IMF content without affecting the quality of primal cuts, potentially maximizing meat production profits. However, further in-depth-investigation is required to elucidate the fundamental molecular mechanisms underlying IMF formation in muscle to produce high-quality meat.

## Figures and Tables

**Table 1:** The composition of the corn-soybean meal basal diet

	Control^1)^	Probiotic^2)^
Composition (g/kg)	1,000	1,000
Corn	690	689
De-hulled soybean flour	170	170
Full fat soy flour	50	50
Fish meal	25	25
Concentrate^3)^	40	40
Lard	25	25
Probiotic supplements	-	1
Calculated value		
ME (kcal/kg)	3,420	3,416.6
Crude protein (%)	15.4	15.4
Ether extract (%)	6.2	6.2
Ca (%)	0.55	0.55
Total phosphorus (%)	0.5	0.5
Lysine (%)	0.94	0.94
Methionine (%)	0.32	0.32
Threonine (%)	0.82	0.82

1) Control (basal diet).

2) Probiotic (control+1×10^9^ CFU/kg *Bacillus amyloliquefaciens*).

3) The concentrate provided the following nutrients per kilogram of the complete diet: 6,480 IU vitamin A as vitamin Aacetate, 2,800 IU vitamin D3 as D-activated animal sterol, 26 mg IU vitamin E as alpha tocopherol acetate, 2 mg vitamin K3 as menadione dimethylpyrimidinol bisulfte, 50 mg thiamin as thiamine mononitrate, 4 mg riboflavin, 3 mg pyridoxine as pyridoxine hydrochloride, 0.03 mg vitamin B12, 23-mg D-pantothenic acid as calcium pantothenate, 20 mg niacin, 1.2 mg folic acid, 0.2 mg biotin, and 300 mg choline as choline chloride. Also provided the following quantities of minerals per kilogram of the complete diet: 95 mg Cu as copper sulfate, 200 mg Fe as ferrous sulfate, 0.35 mg I as potassium iodate, 30 mg Mn as manganese sulfate, 0.30 mg Se as sodium selenite, and 100 mg Zn as zinc sulfate.

**Table 2:** Primers designed for real-time quantitative PCR analysis

Gene	Primer pairs	Annealing (°C)	Product length (bp)
*LPL*	Forward: 5′-GGCCAAATGACACATCTTC-3′ Reverse: 5′-GGCTACGGCTAAACTTACT-3′	60	141
*CD36*	Forward: 5′-GTCAACCTATTGGTCAAACC-3′ Reverse: 5′-CATTTCTGCCTTCTCATCAC-3′	60	123
*FABP3*	Forward: 5′-TTCAAGCTGGGAGTGGAGTT-3′ Reverse: 5′-TCGTAAGTGCGAGTGCAAAC-3′	60	197
*FABP4*	Forward: 5′-GTGGTGGAATGTATCATGAAAGG-3′ Reverse: 5′-ACATCCAACAGAGTGTTGTAGAG-3′	60	108
*SLC27A1*	Forward: 5′-CCATCGGCTTCCTTGAT-3′ Reverse: 5′-GCTACTGGCCTCAATGT-3′	60	120
*PNPLA2*	Forward: 5′-GCCTCTCTACGAACTCAAG-3′ Reverse: 5′-TGAACTGGATGCTGGTG-3′	60	125
*PPARγ*	Forward: 5′-GACAGACCTCAGACAGATTG-3′ Reverse: 5′-GGTGAACGTGGACTTCTT-3′	60	138
*FASN*	Forward: 5′-CGCGGCCTTGAAATTTACTG-3′ Reverse: 5′-GCACACGTGCACTTTAATCG-3′	62	126
*PLIN2*	Forward: 5′-CATTGCCAACACTTACGC-3′ Reverse: 5′-AGTAGTCGTCATAGCATCTT-3′	60	136
*CPT1β*	Forward: 5′-CGTCTCCAGCAAGTTCTCAA-3′ Reverse: 5′-AAGGGCATCTGGTGTTTCTC-3′	62	141
*GAPDH*	Forward: 5′-GTGACACTCACTCTTCCACTT-3′ Reverse: 5′-CCTGTTGCTGTAGCCAAATTC-3′	60	107

PCR, polymerase chain reaction.

**Table 3:** Effects of probiotic supplementation on growth performance of finishing pigs

Traits	Dietary treatment^1)^		p-value

	Control	Probiotics	
Body weight (kg)			
At start	77.51±3.51	77.39±3.17	0.92
Day 14	85.98±5.93	88.05±4.29	0.27
Day 28	96.08±6.36	99.09±4.44	0.13
Day 42	104.55±6.94	109.59±5.29^*^	0.03
Day 56	111.89±6.74	118.15±5.19^*^	0.01
Average daily gain (kg)			
D 0–D 14	0.60±0.33	0.76±0.23	0.13
D 15–D 28	0.72±0.20	0.79±0.16	0.31
D 29–D 42	0.60±0.21	0.75±0.19	0.05
D 43–D 56	0.52±0.15	0.62±0.21	0.19
D 0–D 56	0.61±0.09	0.73±0.08^*^	<0.001

1) Control, basal diet; Probiotics, basal diet+*Bacillus amyloliquefaciens* (1×10^9^ CFU/kg).

* Significantly different compared with the control group at p<0.05 (n = 16).

**Table 4:** Effects of probiotic supplementation on carcass traits of finishing pigs

Traits	Dietary treatment^1)^		p-value

	Control	Probiotics	
Carcass weight (kg)	97.40±2.36	101.49±2.38^*^	0.004
Back fat thickness (cm)	1.86±0.15	2.18±0.31^*^	0.02
Lean meat percentage (%)	55.09±0.82	55.07±0.65	0.97
Fat percentage (%)	7.40±1.02	8.43±1.35	0.11
Bone percentage (%)	13.04±0.68	13.01±0.63	0.91
Loin eye area (cm^2^)	60.08±7.40	61.85±7.20	0.64
Marbling score	1.06±0.18	1.38±0.23^*^	0.01
Shoulder loin (%)	2.55±0.18	2.57±0.24	0.88
Loin (%)	5.29±0.38	5.35±0.16	0.72
Belly (%)	4.67±0.32	4.90±0.41	0.22
Tenderloin (%)	0.62±0.06	0.63±0.04	0.61
Ham (%)	8.70±0.27	8.46±0.41	0.19

1) Control, basal diet; Probiotics, basal diet+*Bacillus amyloliquefaciens* (1×10^9^ CFU/kg).

* Significantly different compared with the control group at p<0.05 (n = 8).

**Table 5:** Effects of probiotic supplementation on meat quality of finishing pigs

Traits	Dietary treatment^1)^		p-value

	Control	Probiotics	
pH_48h_	5.69±0.12	5.67±0.09	0.71
L^*^	51.58±2.23	52.86±1.70	0.22
a^*^	5.18±0.62	5.60±0.27	0.10
b^*^	4.85±0.41	5.47±0.43^*^	0.01
Water holding capacity	54.65±2.79	52.49±1.76	0.08
Crude fat	5.02±0.57	6.26±0.64	0.17
Cooking loss (%)	0.38±0.02	0.36±0.05	0.17
Drip loss (%)	1.12±0.71	1.07±0.62	0.87
Shear force (N)	80.16±11.14	58.58±8.96^*^	<0.001

1) Control, basal diet; Probiotics, basal diet+*Bacillus amyloliquefaciens* (1×10^9^ CFU/kg).

* Significantly different compared with the control group at p<0.05 (n = 8).
